# Supplementary material for: Direct evidence that twisted flux tube emergence creates solar active regions
Source: Nat Commun. 2021 Nov 16;12:6621. doi: 10.1038/s41467-021-26981-7 (PMC8595608; doi:10.1038/s41467-021-26981-7)
Supplement: Supplementary file 1 — Supplementary Information [file 41467_2021_26981_MOESM1_ESM.pdf]

Direct evidence that twisted flux tube emergence creates solar active regions

Supplementary Information

As further evidence of the robustness of magnetic winding in correctly identifying the underlying topology of an emerging flux tube, we present details of a simulation in which a flux tube emerges in a manner more complex than the bipolar cases considered in the main text.

Due to the similarity of the simulation compared to that described in a previous study[32] and the description given in Methods: Numerical simulation details, we only highlight the main differences here. The simulation is first run without a magnetic field until a convection zone develops fully. Then, a magnetic flux tube is inserted 3.4 Mm beneath the base of the photosphere. The tube, with a radius of 595 km, is composed of two parts: a globally twisted field and a mixed helicity perturbation. The constant global twist is 0.3 radians over a distance of 170 km and the tube has an initial axial field strength of 9.1 kG. The mixed helicity perturbation, $\mathbf{B}_{\mathrm{mh}}$, is given by

$$\mathbf{B}_{\mathrm{mh}} = \sum_{i=1}^{2} k_i \exp[-(x - x_0)^2 - (z - z_0)^2 - (y - y_i)^2]((z_0 - z)\mathbf{e}_x + (x - x_0)\mathbf{e}_z),$$

where, in the non-dimensional units described in Methods: Numerical simulation details, $k_i = (1, -1)$, $y_i = (5, -5)$, $x_0 = 0$, $z_0 = -20$ (the dimensionless starting height of the flux tube axis). This mixed helicity perturbation, added to the twisted tube, changes the local field line complexity at the locations with centres (x_0, y_i, z_0), $i = 1,2$. The net twist (or helicity) added by $\mathbf{B}_{\mathrm{mh}}$ to the flux tube is zero.

The tube rises due to buoyancy and becomes deformed quickly by convection. The mixed helicity perturbation plays no noticeable role in the subsequent evolution, which is dominated by the deforming effects of convection. As the flux tube rises to the photosphere, it develops a serpentine geometry and this results in different parts of the tube emerging at slightly different times, referred to here as partial emergences. Supplementary Figure 1 shows an isosurface of the B_y component of the magnetic field, just before a partial emergence.

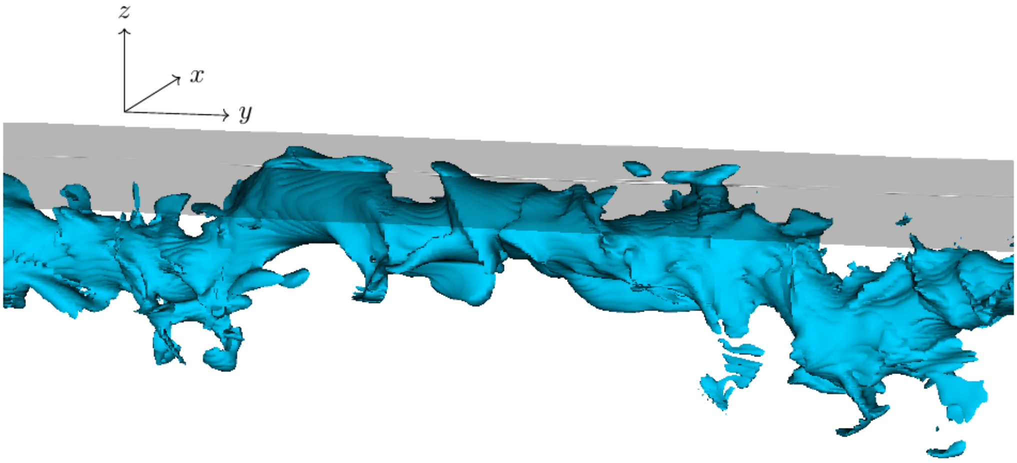

Supplementary Figure 1: Partial emergence. An isosurface (cyan) of the B_y component of the emerging magnetic flux tube. The x-, y- and z-directions are indicated. The tube has a serpentine geometry with different parts emerging at different times. The grey slice shows B_z at the base of the photosphere, through which parts of the tube begin to emerge.

Supplementary Figure 2 shows the total winding accumulation evolving in time.

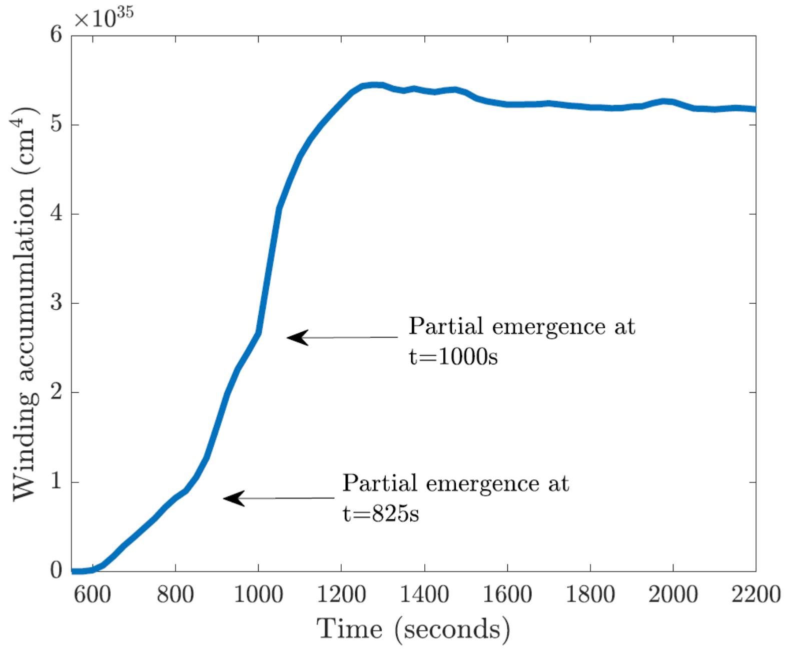

Supplementary Figure 2: Magnetic winding accumulation. The total magnetic winding accumulation with some partial emergences indicated.

There is a clear rise and plateau signature, indicating that the magnetic winding is correctly identifying the underlying field line topology – that of a twisted flux tube. The partial emergences, as indicated in Supplementary Figure 2, do not strongly affect the overall rise and plateau signature as they occur within a short time of each other. If the partial emergences were to be separated by longer times, they would each have a rise and plateau signature. This can be seen to a limited extent in Supplementary Figure 2.

Supplementary Figure 3 shows a magnetogram of B_z at the base of the photosphere at $t = 1800$ s, once the magnetic winding has reached its plateau. It is clear from this figure that there is no simple bipolar structure. Despite this, the magnetic winding is robust enough to detect that the underlying magnetic field has the field line topology of a twisted flux tube.

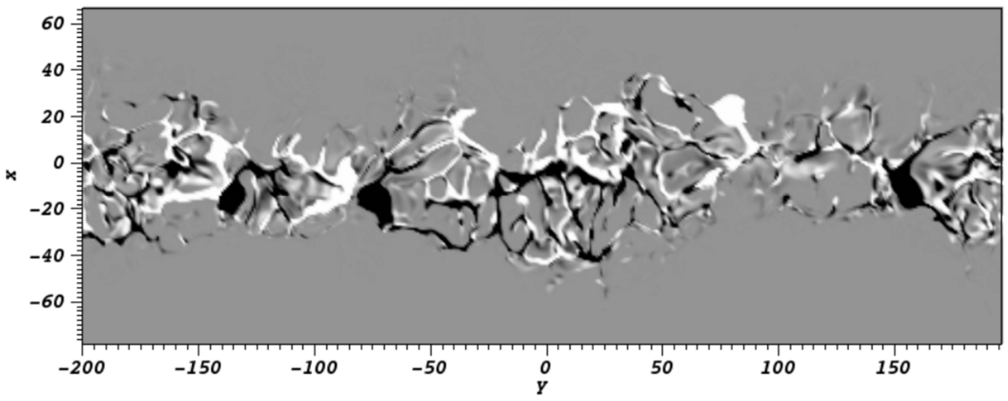

Supplementary Figure 3: Magnetogram. A magnetogram of the B_z component of the magnetic field at the base of the photosphere at $t = 1800$ s. Black indicates negative B_z and white indicates positive B_z. Distances (X and Y) are shown as dimensionless lengths (scale by 170 km to produce physical lengths).
